# I know i’m not meant to ‘diet’ but is it ok to lose a few pounds while pregnant?: a qualitative analysis of Mumsnet discussion forum posts to understand women’s experiences of and views on weight and weight management while living with excess weight during and after pregnancy

**DOI:** 10.1186/s12884-025-08035-8

**Published:** 2025-08-28

**Authors:** Mackenzie Fong, Erin Kelly, Sarah Gregory, Catherine V. Talbot, Maria Raisa Jessica Aquino

**Affiliations:** 1 National Institute for Health and Care Research, Applied Research Collaboration, North East and North Cumbria, Newcastle-upon-Tyne, UK; 2 Population Health Sciences Institute, Newcastle University, Newcastle- upon-Tyne, UK; 3 Department of Psychology, Bournemouth University, Poole, UK

**Keywords:** Qualitative research, Online forum, Pregnancy, Postnatal, Weight management, Weight loss, Obesity, Maternity care

## Abstract

**Background:**

Many women start pregnancy with obesity. It is also common for women to gain excess weight during pregnancy, and many find it challenging to lose this weight after birth. Pregnant and postnatal women may seek weight management support through online discussion forums. This study aimed to explore the experiences and views of weight and weight management in pregnant and postnatal women living with excess weight through analysing discussion forum posts on UK website, Mumsnet.

**Methods:**

Data from Mumsnet discussion forum posted from 7th July 2021 to 7th March 2022 were extracted and included in analysis if they pertained to the experiences of, or views on diet, exercise, or weight management of users with self-reported excess weight during pregnancy or within one-year postnatal. Data were analysed using reflexive thematic analysis.

**Results:**

In all, 113 posts were included. Generally, users had poor awareness of gestational weight gain recommendations. Some reported trying to lose weight during pregnancy (Theme 1: Intentions to lose weight while pregnant: conflicting advice and limited awareness of clinical guidelines). Users shared strategies to manage their weight during and after pregnancy, and specific challenges to this (Theme 2: Approaches and challenges to managing weight during and after pregnancy). Users had mixed experiences of engaging with maternity healthcare professionals and services. Users under consultant-led care reported their concerns about the impact of their weight were dismissed and minimised by healthcare professionals (Theme 3: Mixed experiences of interactions with maternity healthcare services and professionals and unaddressed concerns).

**Conclusions:**

Analysing posts on Mumsnet provided insight into the relatively underreported intention of trying to lose weight while pregnant which is not endorsed by clinical guidelines. It also highlighted the potential for online forums to promote these unendorsed practices. Maternity care professionals should be aware of this and use their encounters with women to discourage intentional weight loss while pregnant. Users under consultant-led care felt that their concerns about their weight were minimised by professionals. Support such as regular weighing was desired but not often provided. Future studies should explore how these aspects of care can be improved.

**Supplementary Information:**

The online version contains supplementary material available at 10.1186/s12884-025-08035-8.

## Background

In the United Kingdom (UK) around one in five women start pregnancy with obesity and this has been increasing over time [1, 2]. Having obesity during pregnancy is associated with greater risk of complications during pregnancy such as gestational diabetes and preeclampsia [3]. There are also implications for longer-term outcomes for women, children, and families. For example, gestational obesity is associated with an increased risk of type 2 diabetes for both the mother and infant, and increased risk of childhood obesity [3–6]. While there is no international consensus on the appropriate amount of gestational weight gain (GWG), guidelines published by the US-based National Academy of Medicine (NAM) [7] (previously the Institute of Medicine) have been adopted by many countries [8]. NAM recommends that women who start pregnancy with overweight or obesity gain less weight than those who start within the recommended body mass index (BMI) range. For example, a woman with a pre-pregnancy BMI of 23 kg/m2 is recommended to gain 11.5–16 kg during pregnancy, while a women starting pregnancy with BMI 29.0 would be recommended to gain between 7 and 11.5 kg. In the UK, the National Institute for Health and Care Excellence (NICE) and the Royal College of Obstetricians and Gynaecologists (RCOG) have not endorsed the NAM guidelines [9] on the basis that the evidence, largely retrospective population-based cohort studies, is insufficient to guide clinical practice. Rather, the UK guidelines recommend that during pregnancy, women are advised to focus on a healthy diet and engaging in physical activity [10, 11]. Women are not routinely weighed unless there is a clinical need, and intentional weight loss during pregnancy is not recommended because of potential adverse effects on the baby [11]. While antenatal support is typically provided by midwives in the UK, women with a pre-pregnancy BMI ≥ 30 are usually offered consultant-led care (provided by a specialist physician) and are referred to a dietitian if needed. Although BMI cut-offs, eligibility criteria for clinical risk factors and service provision varies across National Health Service (NHS) Trusts [12]. Despite these various clinical guidelines, excessive GWG is prevalent in the UK and globally. A 2017 systematic review and meta-analysis including 1,309,136 women found that 47% of women had excessive GWG (above NAM guidelines) [13]. Similarly, a 2019 study analysing individual participant data from 4,429 women from 33 randomised trials found that 36.6% had excessive GWG [14].

Excessive GWG is associated with postpartum weight retention (PPWR) (i.e., retaining the weight gained during pregnancy). On average, women retain around 5–9 kg at one year after birth [15, 16] Studies have shown that among women who start pregnancy with a healthy BMI, 30% will have overweight one year after delivery. Of women who start pregnancy with overweight, 44% are living with obesity one year after delivery [17]. For many women, losing the weight gained during pregnancy is challenging due to childcaring responsibilities, lack of time, domestic disruption, unpredictable schedules, impacts of pregnancy and birth on the ability to exercise, and demands on emotional, cognitive, and material resources [18]. There are no specific NICE guidelines for postnatal weight management; guidelines for the general adult population, including postnatal women, recommend that individuals are provided with referral to behavioural weight management support, diet and physical activity advice and referral for more intensive support as indicated [19].

In the UK, women face barriers to accessing weight management support during and after pregnancy. A 2020 study found that very few maternity healthy weight services in the UK are specifically tailored to women with a raised BMI and provision across geographical areas was patchy [20]. Further, women living with obesity commonly experience weight-related stigma when receiving standard antenatal care [21, 22] and this may deter them from engaging with services and speaking honestly with health care providers [22]. For these reasons, women may choose to self-manage their weight rather than engaging with professional services. This may involve accessing online information and support, including through online communities. Online communities can support pregnant women by facilitating sharing of advice, knowledge, reassurance, and emotional support from peers [23, 24]. Mumsnet is a popular, UK-based website which receives seven million visitors per month [25]. Users can access advice, knowledge, and support on a wide range of topics and users can directly interact with each other via the online discussion forum. Online forums are known to be commonly used by diverse groups of users for elective emotional and informational support [26]. Online forums also provide protected environments to discuss sensitive health issues by affording anonymity and identification with others [27], and the naturalistic nature of online forums provide valuable opportunities for researchers to learn about lived experience [28]. This study aimed to explore the experiences and perceptions of weight and weight management in pregnant and postnatal women living with overweight or obesity through analysing posts on Mumsnet.

## Methods

### Study design

This qualitative study involved analysing content on the publicly available online forum, Mumsnet, to understand the experiences and attitudes of pregnant or postnatal (within one year of giving birth) women with obesity or overweight towards weight management and weight related behaviours (e.g., diet and physical activity). Our approach was informed by the methods presented in a similar study analysing posts on Mumsnet [29].

### Mumsnet

Mumsnet Talk is a popular parent online discussion forum that covers a wide range of topics, many of which are not related to or outside the scope of this investigation. The Talk feature is organised into the following categories: Talk topics, Subtopics, Subjects, and Posts where the content of our analysis was located. Screenshots of the Mumsnet webpage displaying the layout of each category are presented in the Supplementary Material.

### Ethics approval

This study received full ethical approval from Newcastle University Faculty of Medical Sciences Research Ethics Committee (Ref: 14437/2020) and a full risk assessment was conducted prior to undertaking the project. All usernames and any identifiable data were removed to protect the privacy of the individuals posting on the forum during data analysis. While the forum is in the public domain and is publicly accessible, we paraphrased illustrative quotations to minimise the risk of the individual users being identified. Obtaining consent of forum users was not deemed necessary by the Newcastle University Faculty of Medical Sciences Research Ethics Committee as this research involved publicly available online data.

### Data collection, eligibility criteria and data extraction

**Fig. 1:**
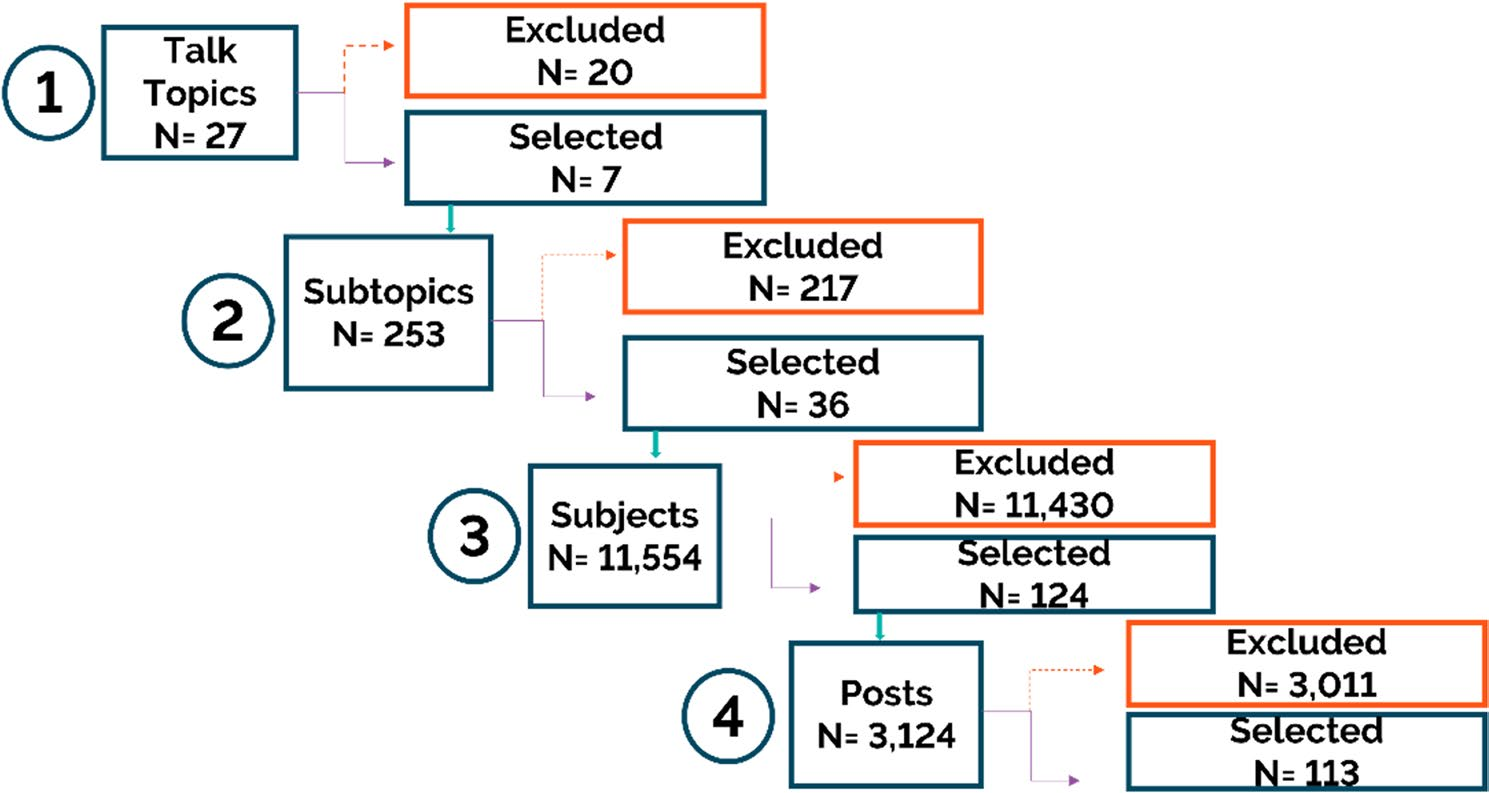
Flow diagram showing the selection of eligible Talk Topics, Subtopics, Subjects and Posts on Mumsnet for inclusion in the study

The freely available web scraping tool Parsehub was used to extract the data from Mumsnet from 7th July 2021 to 7th March 2022, which was exported to Excel. We systematically selected posts from the extracted data for inclusion in the analysis based on the approach of a similar study [29]. This included the use of a priori inclusion and exclusion criteria (reported below) developed to identify and select relevant data. The data collection process followed the forum structure on Mumsnet (Appendix 1). We began by screening the overarching Talk topics on Mumsnet for relevance with reference to the inclusion and exclusion criteria. Examples of relevant Talk Topics included in this study were: ‘Big/slim/whatever weight-loss club’, ‘Health’ and ‘Pregnancy’. The selected Talk topics that were identified as potentially relevant were then searched for their Subtopics. Examples of relevant Subtopics included in this study were “Weight loss chat”, “Weight gain” and “Can I eat. craving all the stuff I’m not allowed.” All Talk topics and Subtopics included in this study are shown in Appendix 2. This process was repeated for each subsequent category (i.e., Subtopic, Subjects, Posts) to identify relevant posts. At the Subjects level, subjects were independently double screened by all authors. Discrepancies in decisions were discussed and resolved at regular team meetings. Figure 1 shows the systematic screening approach. The final dataset was cleaned to remove any personal identifiers such as name, age, location and correct any errors in formatting of text which made the post difficult to read. Usernames were replaced with pseudonyms for use in the illustrative quotes.

### Inclusion and exclusion criteria

To ensure relevant data were captured without manually sieving through and excluding the thousands of posts on the Mumsnet forum, clear inclusion and exclusion criteria were developed alongside restricting the search to the most recent 8-month period (7th July 2021 to 7th March 2022). Posts were considered eligible if they related to experiences of, or attitudes towards diet, exercise, or weight management and living with excess weight while being pregnant or within one year postnatal. Users were considered to have excess weight if their post suggested having a BMI in the overweight or obesity categories; BMIs were calculated where height and weight were self-reported. Posts that featured terms such as ‘plus size’, ‘high BMI’ and ‘fat’ were considered to suggest excess weight and were deemed eligible. Posts that related to an experience beyond the one-year postnatal period or a specific medical condition that required specialised diets (e.g., diabetes) were excluded. Posts which included advertisements or market research were also excluded.

### Data analysis

Reflexive thematic analysis [30] was applied to the data. We followed the 6-phase approach which involved data familiarisation, open and iterative coding of the data, initial theme development, theme refinement, theme finalisation, and write up [30]. Firstly, posts were read multiple times by EK and SG for familiarisation with the data. Next, pilot coding was conducted by all authors on five randomly selected posts to build a foundation of understanding for what was expected of coding. All included posts were then independently double-coded by EK, SG, RA, MF and CT. Meetings took place to compare coding, resolve discrepancies and revise codes i.e., combine/rename. Codes were then used to construct initial themes. These themes were refined and finalised by EK, with support from RA and MF through regular meetings.

## Results

Within 27 Talk Topics, 3,124 posts on Mumsnet were identified as potentially relevant. Of these, 113 posts met the inclusion criteria for inclusion in the analysis. These 113 posts were published by 104 unique users. Of these 104 users, 23 (22.1%) reported living with overweight, 36 (34.6%) reported living with obesity and 45 (43.3%) did not provide information sufficient information to calculate BMI. Of those that were identified as having obesity, ten users (9.6%) were categorised as living with severe obesity (i.e., BMI ≥ 40 kg/m^2^). The greatest proportion of users were within their second trimester of pregnancy (*n* = 24, 23.1%), followed by users within their third trimester (*n* = 22, 21.2%) and users in their first trimester (*n* = 14, 13.5%). Twenty-two women (21.2%) did not state the stage of pregnancy. There were 22 women (21.2%) who reported they were within one year postnatal. Most users did not report how many pregnancies they had experienced (*n* = 74, 75.2%). Seven (6.7%) users stated they had experienced one pregnancy, 14 (13.5%) reported experiencing two pregnancies, six (5.8%) reported experiencing three, and three users (2.9%) reported experiencing four or more pregnancies. Three overarching themes were constructed from the forum data: Theme 1: Intentions to lose weight while pregnant: conflicting advice and limited awareness of clinical guidelines; Theme 2: Approaches and challenges to managing weight during and after pregnancy; and Theme 3: Interactions with maternity healthcare professionals and services and unaddressed concerns.

### Theme 1: intentions to lose weight while pregnant: conflicting advice and limited awareness of clinical guidelines

Users’ knowledge about appropriate weight management during pregnancy were informed by their own experiences of previous pregnancies, self-directed online research, advice from healthcare professionals (usually midwives), conversations with friends and family, and professional health organisations e.g. RCOG, NHS. Generally, users showed awareness of the association between having higher BMI during pregnancy and greater risk of complications such as pre-eclampsia, gestational diabetes and requiring caesarean section. By contrast, users generally had poor awareness about recommended weight change during pregnancy. Specifically, many users reported wanting to lose weight during pregnancy and sought advice from other forum users about whether this could be done safely.

*“I know I’m not meant to ‘diet’ while pregnant but is it ok to lose a few pounds which I can tell are from eating too much*,* not baby?”* (Shanice, BMI “around 27”, pregnant).

*“Before we fell pregnant I lost 2.5st*,* now weighing In at [weight] but I wanted to lose more before falling pregnant for a healthier pregnancy. I totally plan to keep going my weight loss but I’m putting no expectations on myself.”* (Abbie, “plus size”, pregnant).

Users reported experiencing a strong sense of guilt, shame and anxiety associated with their weight and the potential harms to their baby. The main motivation for losing weight during pregnancy appeared to be wanting to reduce the risk of gestational and birthing complications.

*“I’m just wracked with guilt at my weight. I’m the upper end of obese on a BMI chart. I had been trying to lose weight when we decided to try to conceive (I lost about 15lbs) but then that fizzled out when we weren’t falling pregnant and I lost all motivation I’m upset that I may be putting the baby at a higher risk.”* (Shreya, “high side of obese”, pregnant).

Users who were intentionally trying to lose weight during pregnancy often stated that they were not making drastic changes to their diet or exercise and that they would prioritise their baby’s health and development.

*“I’m not dieting but I’m eating really healthily*,* I’m vegetarian so eating lots of fruit and veg*,* lentils greek yoghurt*,* whole wheat carbs*,* eggs*,* etc. This was my diet when I lost weight*,* it was healthy it’s by no means a strict diet and I’ve done lots of research to make sure it’s still healthy while pregnant.”* (Shreya, “high side of obese”, pregnant).

Other users’ responses to posts about attempts to lose weight while pregnant revealed mixed knowledge and views on this topic. Some users showed an awareness of current recommendations to avoid intentional weight loss. By contrast, some users shared their own experiences of successfully losing weight during pregnancy as encouragement to other women, and also suggested that others wanting to lose weight consult a healthcare professional.

*“I don’t think you should try to lose weight while pregnant - i don’t think its even advisable in really overweight people is it? I think the more overweight you are at the start of your pregnancy the less weight you should put on during the pregnancy*,* but i don’t think its an expectation to lose any!”* (Mei Ling, BMI in the late 30 s, postnatal).

*“I’m 32 weeks pregnant and very overweight. This pregnancy I’ve lost about 7lbs just by being a bit more careful I know its not a huge amount but wanted to say it can be done”* (Abbie, “very overweight”, pregnant).

Distinctly, users with higher BMIs who *unintentionally* lost weight during pregnancy, including one who had a recent gastric bypass procedure, were distressed and anxious about the potential impact of unintentional weight loss on their baby. In response, forum users provided emotional support by providing reassurance and occasionally sharing their own experiences of having uncomplicated pregnancies and births despite unintentional weight loss.

*“I am obese and my weight started just under 20 stone I’m now 18 stone and it still seems to be going down although I’m not even trying to lose weight. Obviously I’m obese so I stand to lose the weight but is it safe for baby for me still to be losing weight?”* (Maddie, “obese”, pregnant).

*“My BMI has been 30ish in all my pregnancies*,* and I’ve lost 8-15kg each time. It’s partly because I’ve been sick but mainly it’s been because I have aversions to lots of foods and don’t have a big appetite during pregnancy. All 3 babies have grown well at the moment I’m 31 weeks and I’m 15 kg less than my starting weight!”* (Megan, “BMI of 30ish”, pregnant).

### Theme 2: approaches and challenges to managing weight during and after pregnancy

Users shared their personal experiences of managing their weight both during pregnancy and in the postnatal period, although mostly in relation to the latter. Many users expressed vehemently negative perceptions of their body image, speaking strongly about dissatisfaction with their appearance and experiences of low self-esteem during pregnancy. Users felt that they were being judged by others on their size and appearance. The size and shape of their “baby bump” was a particular source of apprehension around body image.

*“Hi everyone*,* I am plus size and I’m very self conscious….I’m getting so worried about how I will look once I get a baby bump…”* (Priya, “plus size”, pregnant).

The two subthemes below describe the various strategies used to manage weight (subtheme 1) and how pregnancy affected users’ eating and physical activity (subtheme 2).

#### Subtheme 1: strategies for weight management

Approaches to weight management tended to be moderate in nature and included implementing a modest energy deficit (500–1000 kcal per day), time-restricted eating e.g., eating only between 11am-7pm, eliminating soft drinks, reducing snacking, eating regular ‘treats’ to reduce the risk of ‘bingeing’, and engaging in “regular” physical activity including lower intensity activities. Some users were engaging in or expressed interest in behavioural weight management programmes, both during and after pregnancy.

*“I’m on slimming world and plan to drop weight as long as it’s safe or just stay the same weight while I’m pregnant.”* (Dasha, “BMI is just over 40”, pregnant).

Self-monitoring and goal setting were commonly used to support behaviour change. For example, users reported using smart watches to monitor their physical activity levels to promote exercise. Self-weighing in the postnatal period was also reported.

*“The other day I downloaded fitness pal to count calories. I wear an Apple Watch so track my activities on that. I like to burn 520 calories a day. That’s what I aim for each day.”* (Grace, BMI 26, postnatal).

For some users, the desperate desire to lose weight after pregnancy was accompanied by strong dissatisfaction with their appearance, and a shift in their pre- and post-pregnancy self-identity.

*“But my shape has totally changed post pregnancy*,* my hips and ribs are wider and my usually modest boobs are HUGE which could mean I’m never going to be in my size 10 s again!”* (Ayesha, BMI 25.6, postnatal).

*“During this pregnancy at 30 weeks I’ve put on 19 pounds. I look and feel ginormous. I actually cry each morning because of my size. I’m scared I will never look or be attractive again and people now just think of me as a heavy girl. My face my legs and arms. All MASSIVE. I’ve become a hermit because of it I don’t leave the house unless I absolutely have to. Anyone else feeling the same?”* (Dani, BMI 26, pregnant).

#### Subtheme 2: effect of pregnancy on eating and physical activity can impact weight management

During pregnancy, users experienced a range of symptoms that affected eating and physical activity behaviour. These included cravings, heartburn, sickness and nausea, fatigue, pelvic pain, food aversions and changes in appetite. These symptoms had varying effects on users’ eating behaviours and weight. For example, in some women, nausea was associated with reduced dietary intake and was perceived to contribute to weight loss; however, other users reported overeating energy-dense foods that they could tolerated to alleviate nausea, and this was perceived to contribute to weight gain.

*“I did gain a lot of weight in the early stages with awful carb loading with nausea and bmi was 32 at booking.”* (Evelina, BMI 32 at booking, pregnant).

Experiences of appetite appeared to vary between users and within users, and across different stages of pregnancy. Some users experienced intense hunger, while others reported an absence of hunger.

*“I had a big appetite in the 2nd trimester… it did drop off when I got into the 3rd trimester so it’s much easier to eat reasonably now”* (Barbara, BMI 32 at booking, pregnant).

*“But I’m also really concerned by the lack of weight gain. I’m not feeling hungry and sometimes I’m just forcing myself to eat.”* (Claire, “overweight”, pregnant).

While some users expressed concerned about the effect that reduced dietary intake may have on the health of their baby, others viewed incidental weight loss as potentially beneficial to their longer-term weight management.

*“If you’re like me and you get heartburn in the third trimester*,* you might find your weight is a lot less after the birth than before. If so*,* the trick is to harness it and not stack it back on like me*,* both times”* (Daisy, BMI “well over 30”, postnatal).

Engaging in physical activity during pregnancy was hampered by fatigue and physical discomfort such as pelvic pain or pain associated with diastasis recti (separation of the abdominal muscles). One user who experienced several miscarriages was anxious about the potential harms of exercise to her baby.

*“But when I have somewhat exerted myself eg a sudden fast walk or an on call at work (it can be quite physical work in the nhs)*,* I have bled. And not just spotting. I mean overflowing bright red through pads. Which is really stressful and upsetting.”* (Julia, “just over BMI of 30”, pregnant).

Many users reported wanting to lose weight after pregnancy but experienced challenges to this including a lack of energy and cognitive resource to prepare food, disruptions to daily routines and practical aspects of caring for a newborn and potentially other children.

*“It’s really difficult to find the time. We have a toddler too by the time both kids are down and settled we need a quick dinner and I’m in bed by 9”* (Lydia, “I’ve never been as big”, postnatal).

*“What’s even worse im not even doing any exercise as I just always feel shattered. Not good!”* (Zoe, BMI 32, pregnant).

Several users who wanted to lose weight in the postnatal period felt unsure of how to implement an energy-restricted diet without compromising their breastmilk production.

*“I have gained a lot weight while pregnant and ideally would like to lose weight as soon as possible after birth. I understand that cutting out too many calories will impact breast milk quality/quantity. Did you successfully lose weight while breastfeeding and what approach did you take?”* (Agata, “gained significant amount of weight whilst being pregnant”, pregnant).

#### Theme 3: mixed experiences of interactions with maternity healthcare services and professionals and unaddressed concerns

Users shared their experiences and expectations of engaging with maternity services and professionals as expectant mothers with excess weight (Subtheme 1). Those who were receiving specialist maternity care because of their weight shared their experiences of this (Subtheme 2).

#### Subtheme 1: expectations and experiences of maternity healthcare services and professionals

Users discussed their actual and anticipated experiences of engaging with maternity services and healthcare professionals including medical consultants, midwives, dietitians, and nutritionists, during their pregnancy. Some users who had not yet engaged with healthcare services or professionals expressed apprehension and uncertainty about what to expect and were sceptical about the usefulness of the service.

*“Do I have to book in to see the dietician? I can’t stand having to take work off to be chastised on healthy eating when I know what to eat and what not to eat and to eat as healthy as possible for this baby. Can I just carry on?”* (Harriet, BMI “just over 40”, pregnant).

Conversely, other users were eager to receive nutrition advice and additional support for weight management and acknowledged the value of engaging with a qualified professional.

*“As a plus size I would love the chance to get guidance on eating during pregnancy. I have good knowledge of healthy eating*,* that’s not the reason I eat unhealthy foods. Having support for healthy eating is beneficial from anyone and absolutely more from a dietitian than slimming world. I think it’s very hard to get the balance during and watching your weight and diet as well as the baby’s is complex.”* (Yuan Jiao, “plus size”, pregnant).

A particular source of apprehension for many users was whether their weight would affect the quality of their pregnancy scans. Users who had their scans shared their experiences which were a mixture of positive and negative.

*“The midwives just told me that baby was positioned awkwardly and that was that. I didn’t get any pictures and had no nuchal screening. Later*,* my records were updated saying they couldn’t scan me due to BMI.”* (Lani, BMI 46 at booking, pregnant).

Users who had engaged with healthcare services and professionals, including one who attended a specific maternity weight management programme, found these services and interactions largely positive, even despite initial scepticism. Users reported that healthcare professionals provided reassurance, were sensitive about users’ weight, and helped with pregnancy specific challenges.

*“I was skeptical about it at first but glad I persisted because i then had terrible sickness and she gave me suggestions of things to try and eat. and also reassurance as being sick made me quickly lose weight weight.”* (Megan, “plus size”, pregnant).

Some users reported negative encounters with health care professionals which were generally related to the use of language around weight and weight management.

*“I’ve got several rude and unnecessary comments related to my weight.”* (Hannah, BMI 37, pregnant).

Conversations around weight were psychologically triggering for one user who had a history of an eating disorder and mental illness.

*“During this conversation the midwife said I was clinically obese and a risk to the baby. I told her that this was very triggering for me (give I had an eating disorder 5 years ago*,* and focusing on weight triggered depression after last baby). I understand why extra care is needed*,* however I had no complications in my last pregnancy and I had a natural birth (bmi 35) and there was never a mention of a consultant.”* (Khari, BMI 37, pregnant).

#### Subtheme 2: unmet needs of women receiving specialised care for higher BMI pregnancies

Aside from receiving additional surveillance such as extra scans and tests during pregnancy, most users felt that their care was mainly unchanged. Users reported that their weight was rarely discussed, nor were they weighed during their care contacts.

*“At my 8 week booking appointment I was referred for consultant led care. So far I’ve had 1 phone call with the consultant & there’s been hardly any mention of my weight*,* definitely no one is pressuring me to lose weight. They were mainly worried about gestational diabetes*,* which I did get diagnosed with at 26 weeks. Overall*,* I feel taken care of well & assured that’s additional care is available.”* (Rose, BMI 36, pregnant).

A few users received brief advice about the risks of a higher BMI during pregnancy, and some were advised not to intentionally attempt to lose weight during pregnancy.

*“Very quick appointment - they spoke about the risks of a high BMI*,* although they told me not to try to lose weight while pregnant.”* (Laura, BMI 35.2 at booking, pregnant).

While some users appeared indifferent to the lack of weight-specific care and were glad that they were not being pressured to lose weight, many reported that their anxieties and concerns regarding their weight were unacknowledged and unaddressed by healthcare professionals. These concerns related to the risks of a higher BMI pregnancy but also to unintentional weight loss during pregnancy. Users who voiced their anxieties felt that their concerns were largely dismissed and minimised by healthcare professionals who did not offer reassurance, information or support.

*“I’m 34 years old & 17 weeks pregnant and I’ve lost 17 pounds! At conception I weighed 237 anyone else in a similar position? No sickness from 12 weeks. OB doesn’t seem worried? But I am! (OB = obstetrician)”* (Helen, “weight at conception was 237”, pregnant).

*“At my 20 week scan they said baby is average size and not to worry*,* and I’m being managed by a consultant because of my age*,* weight and the fact that my son was SGA (small for gestational age) I told the doctor about the weight loss when I saw them but they didn’t seem too fussed. I’ve been given aspirin to take every day and I’m stating clexane in a few weeks based on my bmi*,* but since I was first weighed I’ve lost 6 kg and they haven’t wanted to weigh me since.”* (Ashleigh, “overweight”, pregnant).

Avoiding discussions about weight was perceived to be because health care professionals did not want to cause women offence or embarrassment.

*“I’m have a bmi of 37 and I’m 35 weeks pregnant. My bmi was mentioned to me in any scans*,* I’m guessing because a lot of women would be offended.”* (Elena, BMI 37, pregnant).

Some users indicated they wanted to have their weight measured, however, this was refused by their health care team.

*“During my last pregnancy they refused to weigh me and said that it really didn’t matter. Although I was seeing a consultant for high BMI of 35*,* I was also under the consultant for other medical issues not related to weight. They hardly referred to my weight at all and there was no difference to my care specifically because of my weight issues.”* (Una, BMI 37, pregnant).

## Discussion

Mumsnet provides expectant and new mothers with a social network to discuss their concerns and share experiences about weight change and weight management, exchange advice, and familiarise themselves with the pregnancy experience (for first-time pregnancies). Users in our study showed some awareness of the link between elevated weight and greater risk of pregnancy and birthing complications including preeclampsia, diabetes, and caesarean section. Our findings align somewhat with a previous study in 364 pregnant women (47% with overweight or obesity) whereby 94% of women believed that excess GWG or obesity would be associated with increased pregnancy complications; however, study participants’ knowledge of the specific nature of these risks was poor [31]. In agreement with several published reviews of qualitative studies on the topic [21, 32], we found that Mumsnet users were generally unaware of GWG guidelines; specifically, they showed poor awareness of recommendations to avoid intentional weight loss. The confusion around appropriate GWG may stem from an absence of clear guidance on recommended GWG in the UK, such as NAM guidelines. Although, a meta-synthesis of qualitative research found that women from other developed countries including those that have adopted NAM guidelines (Canada and United States of America) also did not know the recommended weight gain in pregnancy used in their country [33]. To the authors’ knowledge there is at least one NHS Trust in the UK that has adopted NAM guidelines on GWG whereby women are routinely weighed and advised on BMI-specific GWG ranges. An evaluation of the impact of this model of care on women’s GWG and understanding of healthy weight gain would help inform policy and future service delivery in the UK.

Users reported being anxious about the impact of excess weight on their baby, and because of this, they reported intentions to lose weight during pregnancy. In response, some users provided encouragement by sharing their experiences of intentionally losing modest amounts of weight during pregnancy. Our finding highlights the potential role of Mumsnet to promote misinformation and unendorsed weight management practices, and the unanticipated (negative) consequences of social support, despite positive intentions. Intentional weight loss during pregnancy is not recommended by professional health bodies (i.e., NAM, RCOG, NICE) during pregnancy based on evidence showing that gestational weight loss is associated with increased odds of having a small for gestational age baby (compared to GWG within guidelines) and a lack of information on preterm birth [34]. There are few studies exploring women’s intentions to lose weight during pregnancy. An analysis of cross-sectional US data from approximately 2,500 women aged 18–44 years found that 7.5% of pregnant women were trying to lose weight [35]. This was higher in women with a higher BMI, with diabetes, hypertension and those who had received weight management advice [35]. These data were collected in 2003, and it is likely that the proportion of pregnant women trying to lose weight is now higher given the rise in obesity prevalence since publication of this study. Importantly, many women who reported to lose weight during pregnancy emphasised their intention to prioritise their baby’s wellbeing, in agreement with previous studies [32].

That users were unaware of recommendations to avoid intentional weight loss during pregnancy may be partly due to poor, and inconsistent provision of weight management information and support by maternity healthcare providers. In our study, Mumsnet users, including those under consultant-led maternity care for their weight, reported that their weight was rarely discussed during their antenatal visits. This is consistent with findings of several published studies observing that provision of weight management information and support was absent, inconsistent, and vague (e.g. “don’t gain too much weight”) [22, 36]. In our study, users under consultant-lead care were eager to discuss with health care professionals their anxieties about their weight (including unintentional weight loss) and the impact on their baby, however, their concerns were minimised and dismissed. Research suggests that weight management is not routinely discussed with pregnant women with obesity because health care professionals do not feel comfortable or confident having conversations about weight and try to avoid them to minimise offence or embarrassment [37]. Further, in our study, users reported that they wanted to be weighed regularly. Previous research also shows that pregnant women with obesity wanted to be weighed regularly [38, 39] and that this would serve as a motivational tool [39].

In agreement with previous research [40, 41], we identified specific challenges to weight management spanning the pregnancy and postnatal period including physical and mental fatigue, changes to appetite and food preferences, fear of harming the baby during pregnancy through exercise, physical discomfort, and domestic disruptions and lack of time due to child caring responsibilities in the postnatal period [36]. Users also expressed uncertainty about trying to lose weight through energy restricted diets while breastfeeding in the postnatal period; current UK guidelines state that gradual weight loss will not adversely affect the ability to breastfeed or the quantity or quality of breast milk [11]. Some Mumsnet users suggested that others wanting to manage (and lose) weight during or after pregnancy engage with a structured community weight management service which are provided by local councils in the UK. However, very few community weight management services are tailored to suit the specific needs of pregnant and postnatal women [42], and of those available, most need to be privately funded. As maternal obesity contributes significantly to population-level obesity, there is a need to determine how weight management support can be tailored to address the specific challenges faced by pregnant and postnatal women, and how this support can be delivered at scale and equitably, such as embedding supporting into routine primary and community care contacts in the postnatal period [43], for example, via statutory child health visiting contacts in the UK.

In our study, some users showed an unclear understanding of maternity healthcare professionals’ roles, particularly dietitians. Uncertainty about the role of health professionals sometimes included preconceived negative views, which made users reluctant to accept support. We observed that users who had engaged previously with health care support and professionals shared their experiences of this on the forum, including the benefits they had received, as reassurance to others. This highlights the potential role of women who have engaged with pregnancy services in addressing misconceptions about healthcare professionals and encouraging other women in similar positions to seek and/or access professional support. This is supported by a study assessing the role of online mental health communities, one of the main themes identified was ‘transition to further support’ which highlighted how users provided each other with the confidence and direction to seek further support [44]. Users also shared negative experiences of engaging with healthcare professionals and services on the forum, including receiving degrading or insensitive comments about their weight. As many users expressed intense dissatisfaction with their appearance and body image, insensitive language about weight may have significant implications such as deterring women from engaging with healthcare services. Although limited, studies reporting on overweight or obese pregnant women’s perceptions of their bodies align with our findings. Sui et al. found that 29% of overweight and 70% of obese were dissatisfied with their weight and 30.4% and 68% for body shape, respectively [45]. They also found a statistically significant relationship between those who experienced higher dissatisfaction and higher gestational weight gain (*R*^2^ = 0.133; *p* = 0.018).

### Implications and recommendations

In the absence of evidence endorsing gestational weight loss, there is a need to enhance women’s awareness of avoiding intentional weight loss. This could be achieved through healthcare professionals (i.e., GPs and midwives) discussing this with women in the early stages of pregnancy. As women with obesity, especially those under consultant-lead care, may be anxious about the impact of their weight on their baby, they should be provided with opportunities to discuss this during interactions with healthcare professionals and offered to be weighed. When discussing weight and weight management, healthcare professionals need to be sensitive to women’s body image; further research is needed to determine what language and approaches are most appropriate. We observed that Mumsnet allowed women to clarify the role of maternity healthcare professionals and encouraged engagement with health care services. Future studies could assess the feasibility of online peer support groups to overcome barriers to engagement with professional weight management support before, during and after pregnancy. Lastly, future studies could use our findings to inform future research interviews or questionnaires.

### Strengths and limitations

We used a systematic approach using a priori eligibility criteria to identify posts that were highly relevant to the research question. Data screening and coding took place independently and in duplicate, with meetings to discuss and resolve discrepancies. This approach added to the trustworthiness of presented data. We used data from Mumsnet, which is an unregulated and anonymous forum and offers a space to explore women’s candid attitudes. This approach is particularly valuable for sensitive/stigmatised topics which participants may be less likely to disclose in traditional qualitative data collection methods such as interviews. The forum also provided a platform for non-prompted discussions where women instigated topics of importance to them, as opposed to a researcher-led interview. The methods used in this study allowed us to make a novel contribution to research on weight management during pregnancy; very few studies have explored women’s attitudes towards intentional weight loss during pregnancy, nor have discussed unanticipated consequences online forum i.e., promote misinformation and unendorsed weight management practices.

Our findings should be viewed considering its limitations. Firstly, as we based our inclusion criteria on women’s self-reported weight, and even subjective descriptions of their weight, it is possible that we have inadvertently captured views of women who have a BMI in the recommended range who may have distorted body image or disordered eating; this may affect the study’s findings. Secondly, using data from Mumsnet means that data may not be representative as users tend to be university educated and have an above average household income [29]. Women with lower income and education may face different barriers to weight management (e.g., food insecurity [46]), and therefore, share different experiences to those identified in the current study. Also, as Mumsnet is online, it requires internet access and ability to communicate in writing, those from lower socioeconomic backgrounds or lacking in formal education may be under-represented. Also, as the forum is online, this prevents the researchers from interrogating posts to expand upon or clarify meanings, therefore, there is a risk of misinterpretation of data. Not being able to interact with participants meant that we could not determine all of their characteristics i.e., where users lived during and after their pregnancies; users’ height and weight, users’ ethnicity. Future research should explore the inclusion of women from different socioeconomic and cultural backgrounds, particularly those from racialised and marginalised communities, and appropriate demographic data collected to developed nuanced and rich understandings of lived experience of weight change during and after pregnancy.

## Conclusions

Mumsnet forum data provided insight into the relatively underreported intention to lose weight during pregnancy which is not endorsed by clinical guidelines. It also highlighted the potential for online forum to promote unendorsed weight management practices. Maternity health care professionals should be aware of this and use their encounters with women to discourage intentional weight loss while pregnant. Even though they expressed their anxieties about their weight, users under consultant-led care because of their weight felt that their concerns were minimised by professionals and additional support for weight such as regular weighing was desired but not commonly provided. Future studies should explore how these aspects of care can feasibly be improved.

## Supplementary Information

Supplementary Material 1

Supplementary Material 2

## Data Availability

The data analysed in the current study are not publicly available to protect the anonymity of the users’ but are available from the corresponding author on reasonable request.
