# Supplementary material for: I know i’m not meant to ‘diet’ but is it ok to lose a few pounds while pregnant?: a qualitative analysis of Mumsnet discussion forum posts to understand women’s experiences of and views on weight and weight management while living with excess weight during and after pregnancy
Source: BMC Pregnancy Childbirth. 2025 Aug 28;25:898. doi: 10.1186/s12884-025-08035-8 (PMC12392490; doi:10.1186/s12884-025-08035-8)
Supplement: Supplementary file 2 — Supplementary Material 2 [file 12884_2025_8035_MOESM2_ESM.docx]

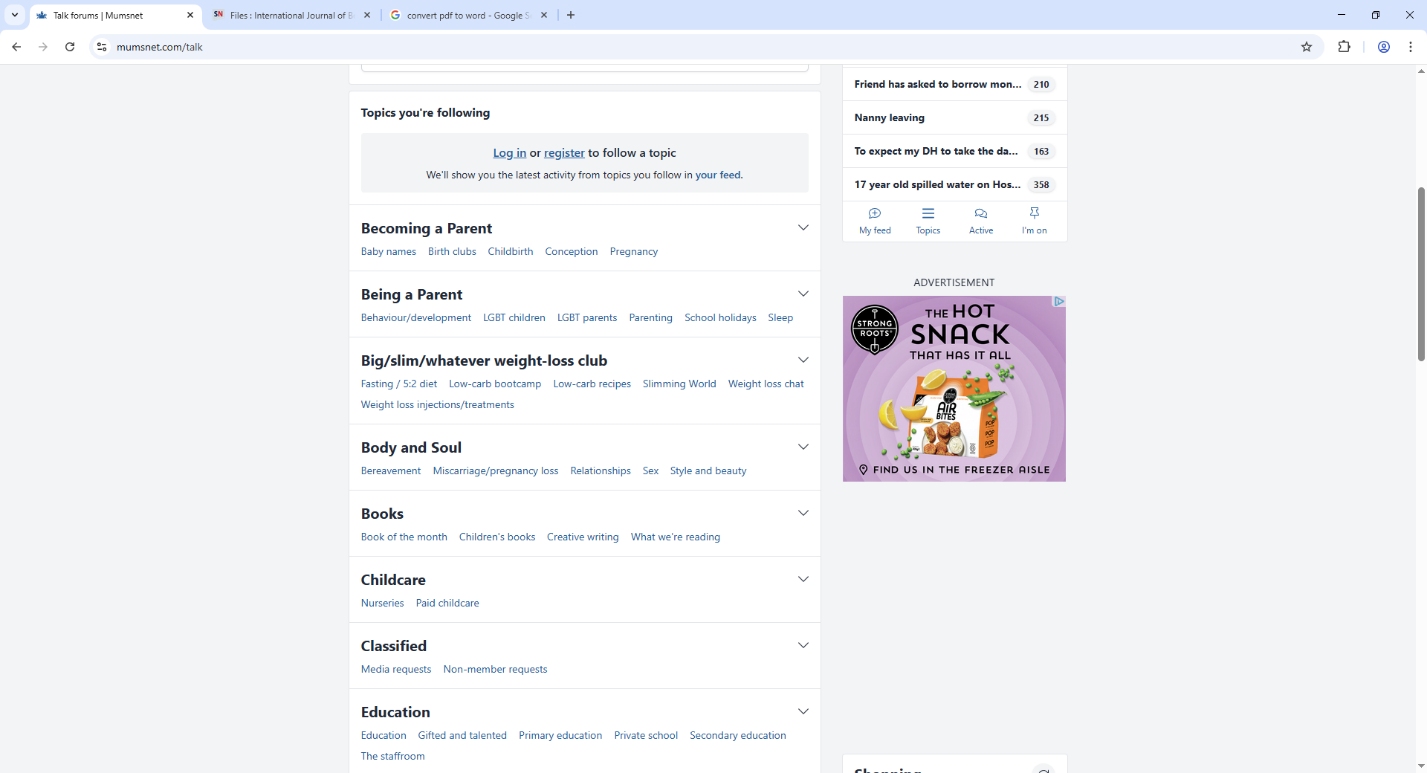
**Appendix 1**

Talk Topic

Talk Subtopics

**Appendix Figure 1.** Layout of Mumsnet ‘Talk’ discussion forum including examples of Talk Topics (e.g., Big/slim/whatever weight-loss club) and Subtopics (Fasting / 5:2 diet; Low-carb bootcamp; Low carb recipes; Slimming World; Weight loss chat; Weight loss injections/treatments).


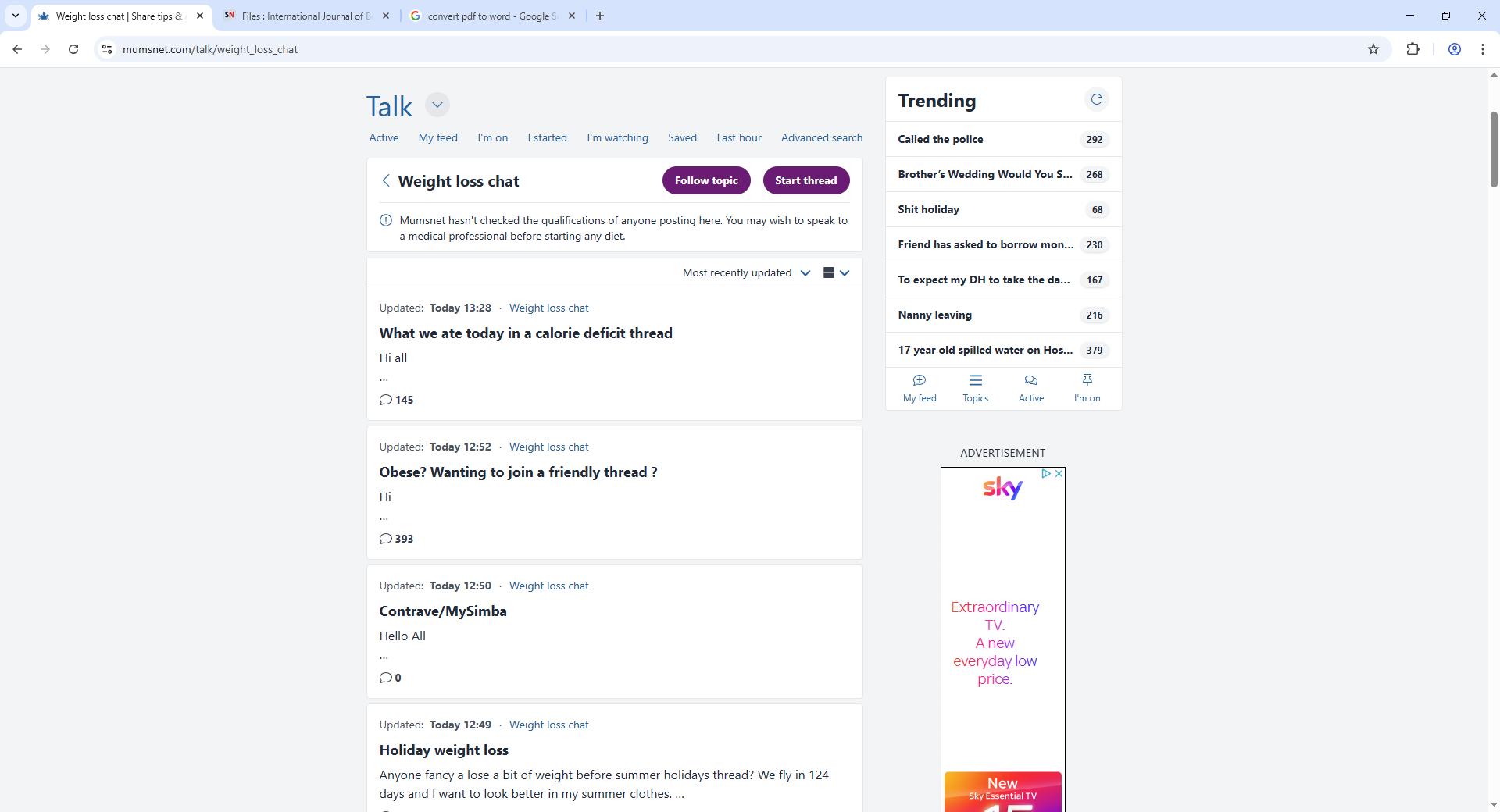


Talk Subjects

Talk subtopic

**Appendix Figure 2.** Layout of Mumsnet ‘Talk’ discussion forum including examples of Talk Subtopics (e.g., Weight loss chat) and Subjects (e.g., Obese? Wanting to join a friendly thread?).


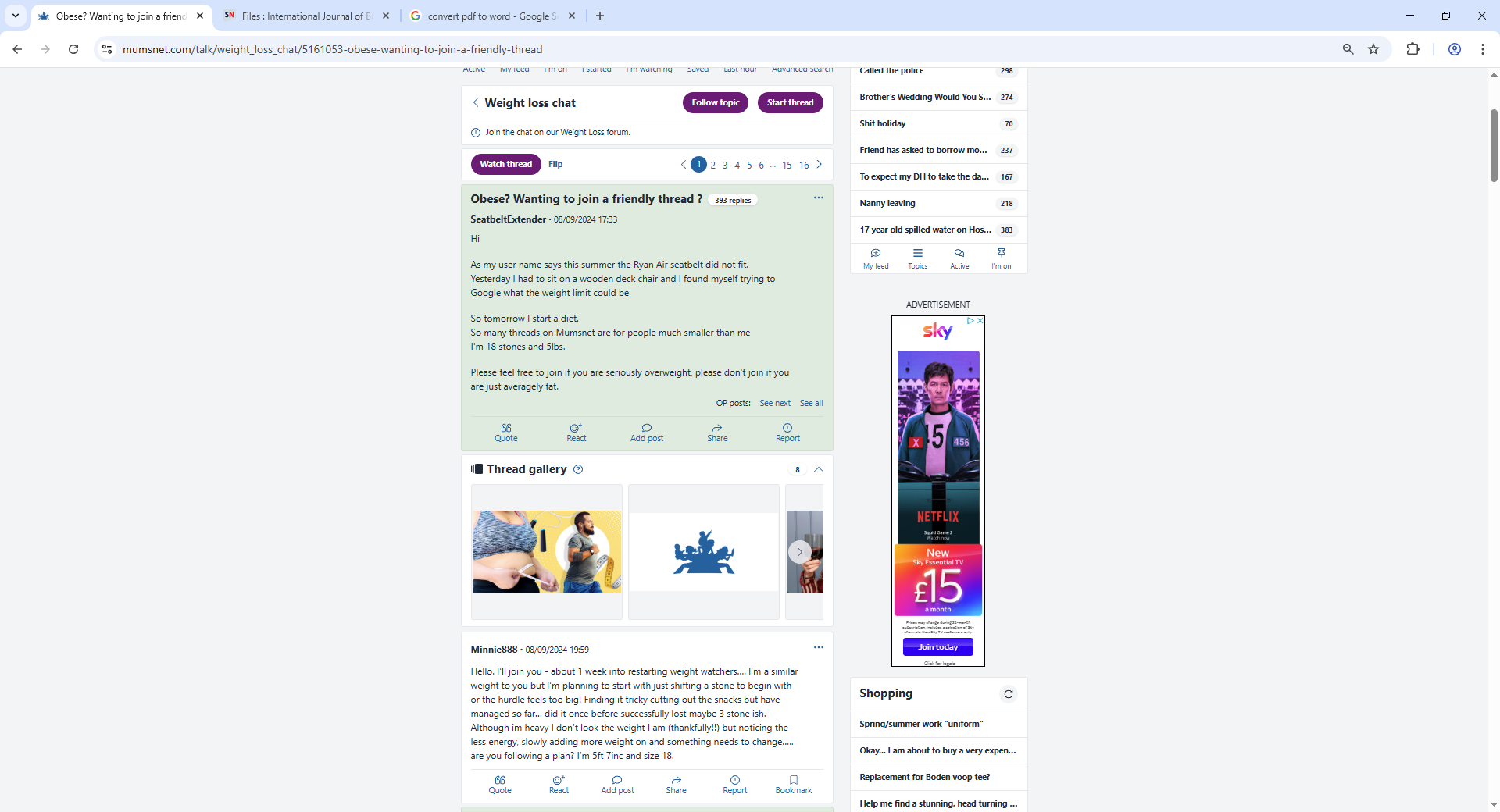


Talk Posts

Which contain

Talk Subjects

Talk Subtopics

**Appendix Figure 3.** Layout of Mumsnet ‘Talk’ discussion forum including examples of Talk Subtopic (Weight loss chat), Talk Subject (e.g., Obese? Wanting to join a friendly thread?) and Talk Posts (the messages and replies posted by users and the unit of analysis used in the study).
